# Application of Machine Learning in Multimorbidity Research: Protocol for a Scoping Review

**DOI:** 10.2196/53761

**Published:** 2024-05-20

**Authors:** Danny Jeganathan Anthonimuthu, Ole Hejlesen, Ann-Dorthe Olsen Zwisler, Flemming Witt Udsen

**Affiliations:** 1 Department of Health Science and Technology Faculty of Medicine Aalborg University Gistrup Denmark; 2 Clinic for Rehabilitation and Palliative Medicine Rigshospitalet Copenhagen Denmark; 3 Department of Clinical Medicine University of Copenhagen Copenhagen Denmark

**Keywords:** multimorbidity, multiple long-term conditions, machine learning, artificial intelligence, scoping review, protocol, chronic conditions, health care system, health care

## Abstract

**Background:**

Multimorbidity, defined as the coexistence of multiple chronic conditions, poses significant challenges to health care systems on a global scale. It is associated with increased mortality, reduced quality of life, and increased health care costs. The burden of multimorbidity is expected to worsen if no effective intervention is taken. Machine learning has the potential to assist in addressing these challenges since it offers advanced analysis and decision-making capabilities, such as disease prediction, treatment development, and clinical strategies.

**Objective:**

This paper represents the protocol of a scoping review that aims to identify and explore the current literature concerning the use of machine learning for patients with multimorbidity. More precisely, the objective is to recognize various machine learning models, the patient groups involved, features considered, types of input data, the maturity of the machine learning algorithms, and the outcomes from these machine learning models.

**Methods:**

The scoping review will be based on the guidelines of the PRISMA-ScR (Preferred Reporting Items for Systematic Reviews and Meta-Analyses Extension for Scoping Reviews). Five databases (PubMed, Embase, IEEE, Web of Science, and Scopus) are chosen to conduct a literature search. Two reviewers will independently screen the titles, abstracts, and full texts of identified studies based on predefined eligibility criteria. Covidence (Veritas Health Innovation Ltd) will be used as a tool for managing and screening papers. Only studies that examine more than 1 chronic disease or individuals with a single chronic condition at risk of developing another will be included in the scoping review. Data from the included studies will be collected using Microsoft Excel (Microsoft Corp). The focus of the data extraction will be on bibliographical information, objectives, study populations, types of input data, types of algorithm, performance, maturity of the algorithms, and outcome.

**Results:**

The screening process will be presented in a PRISMA-ScR flow diagram. The findings of the scoping review will be conveyed through a narrative synthesis. Additionally, data extracted from the studies will be presented in more comprehensive formats, such as charts or tables. The results will be presented in a forthcoming scoping review, which will be published in a peer-reviewed journal.

**Conclusions:**

To our knowledge, this may be the first scoping review to investigate the use of machine learning in multimorbidity research. The goal of the scoping review is to summarize the field of literature on machine learning in patients with multiple chronic conditions, highlight different approaches, and potentially discover research gaps. The results will offer insights for future research within this field, contributing to developments that can enhance patient outcomes.

**International Registered Report Identifier (IRRID):**

PRR1-10.2196/53761

## Introduction

### Background

According to the World Health Organization, multimorbidity is characterized as the coexistence of 2 or more chronic conditions in a single individual. These chronic conditions are often long-term health conditions that require complex and ongoing care [1]. Chronic diseases include both mental and physical health conditions [2]. In the context of multimorbidity, no single chronic condition is necessarily more central than the others, as this could result in designating one condition as the index condition, where a more appropriate term would be comorbidity [2-4].

On a global scale, approximately one-third of adults, and over half of all adults with any chronic condition, already experience multimorbidity [5]. The occurrence of multimorbidity increases with age [5-7], with projections showing a doubling of individuals aged 60 years and older globally before 2050; the number of persons with multimorbidity is also expected to rise dramatically [5,8].

In Denmark, a report from 1 of the 5 regions responsible for delivering health care (Region Zealand) concludes that in 2022, there were approximately 1.2 million people with multimorbidity in Denmark, which corresponds to 26% of the population [9]. This number is expected to increase by 1.4% annually to 1.5 million in 2050 if no efforts are made to improve the population’s health [9].

Multimorbidity is linked to an increased likelihood of premature death [10,11]. This risk escalates with both the quantity of conditions and the particular combinations of chronic diseases [10,11]. Multimorbidity also lowers quality of life, which gets worse as you have more chronic diseases [11-14]. It disproportionately affects individuals with low socioeconomic status, contributing to increased health inequality [5,15-17], and is also associated with more mental symptoms and a greater perception of fragmented care [10,11]. Multimorbidity is also responsible for higher health care expenditure due to longer hospital stays and larger medication consumption [12,18].

There exist several risk factors associated with the development of multimorbidity including sociodemographic factors (eg, age, household income, and education) [5,14], biomedical risks (eg, overweight, high blood pressure, high cholesterol, and genetic predispositions), and health behaviors (eg, physical inactivity, poor nutrition, smoking, and alcohol consumption) [19,20]. Additionally, certain chronic conditions may share common risk factors, or one disease may be a risk factor for another [21].

However, in contrast to patients with single diseases, there is a complex relationship between risk factors and multimorbidity [22]. For example, according to a report from the Australian government [23], there is a correlation between the number of chronic conditions a person has and the likelihood of having multiple risk factors [23].

Nonetheless, without the information regarding the duration of an individual’s exposure to risk factors and the onset of their chronic condition, attributing their chronic conditions solely to the number of risk factors is not possible. This is due to the onset of some chronic conditions possibly motivating people to change their behavior for the better. For instance, when a person is diagnosed with chronic obstructive pulmonary disease (COPD), it may inspire them to quit smoking and thereby reduce the risk of exacerbating COPD and cancer. In contrast, a person diagnosed with COPD will not be motivated to engage in physical activity, which would then increase the risk of, for example, diabetes [19-21].

As a result of the complexity of the risk factors, clinical decisions for patients with multimorbidity are a complicated and challenging task since the health care system is primarily designed to manage patients with a single disease [24].

Understanding the patterns and factors associated with multimorbidity, particularly the modifiable factors, can help contribute to the prevention and treatment of multiple chronic diseases [24,25]. Predictive analytics, such as machine learning, has the potential to solve these kinds of problems [26]. It enables more advanced analysis, automation, and recognition of unidentified patterns [27]. Machine learning enables systems to learn and improve from experience without explicit programming. It involves the process of fitting one or more statistical models to a given data set that contains both explanatory variables and an outcome variable. The models provide coefficients that quantify the relationship between the explanatory variables and the outcome. These model coefficients are applied to predict the same outcome with the same explanatory variables but on new, unseen data. The model’s ability to predict the outcome depends on its capacity to recognize and apply statistical patterns found in the data while it is being trained [27,28].

In health care, machine learning has demonstrated improvements in various tasks, including drug discovery [29], histopathological diagnosis [30], brain magnetic resonance imaging segmentation [31], and disease prediction using electronic health records [32,33]. In the context of machine learning for multimorbidity, there are numerous options and choices when developing an algorithm that are relevant. Combinations of disease groups have the potential to uncover new mechanisms of diseases, develop treatments, develop multidisease clinical strategies, meet the patient’s needs, and manage polypharmacy [34,35]. For instance, Prados-Torres et al [36] have identified 3 common multimorbidity clusters: cardiovascular and metabolic diseases, mental health problems, and musculoskeletal disorders.

Different outcomes have also been addressed in the literature, such as predicting a new disease for patients with multimorbidity [25,37,38], identifying factors for multimorbidity [39,40], predicting rehospitalization [41], and developing a multimorbidity frailty index [42]. Furthermore, a variety of different machine learning models have been used, for example, logistic regression [37,39,40], random forest [25,41,42], neural network [37,40], and network model [38]. It is also evident that a wide range of features have been applied in developing these machine learning algorithms. These features include sociodemographic data [25,41,42], electronic health record data [39,41], self-reported data [40], and medical data [38,39,42], among others.

The literature indicates ongoing research and a growing interest in the field of multimorbidity. However, the type and amount of evidence remain unclear as well as potential gaps in the literature [25]. Therefore, a scoping review was chosen to identify and map available evidence within this field.

### Review Objective

The objective of this scoping review is to explore the existing literature regarding the use of machine learning in the context of multimorbidity. Specifically, it seeks to identify the machine learning models that have been used, the patient groups that have been the focus in various studies, the features, types of input data, the maturity of the algorithms, and outcomes that have been in focus. In doing so, any potential gaps in research or knowledge can be identified.

## Methods

### Ethical Considerations

Ethics approval is not required, as this scoping review does not involve primary data collection from human participants.

### Study Design

The development of this protocol has followed the guidelines from Joanna Briggs Institute [43]. The conduct and reporting of this scoping review will follow the guidelines of the PRISMA-ScR (Preferred Reporting Items for Systematic Reviews and Meta-Analyses Extension for Scoping Reviews) [44]. Furthermore, the review will follow the methodological framework for scoping reviews outlined by Peters et al [45], which provides guidance in the development and execution of a scoping review.

### Search Strategy

#### Overview

To identify appropriate search terms, keywords, and index terms, a preliminary search will be conducted. The initial search will identify text words that can be applied in the subsequent systematic search. The search strategy will be adjusted to the requirements of each database and will include a range of related terms, synonyms, acronyms, and spellings. Furthermore, various search functions, such as thesaurus, Boolean operators, truncation, phrase searching, and free-text searching, will be applied to optimize the searches. The searches will be done in the databases PubMed, Embase, IEEE, Web of Science, and Scopus.

After conducting the searches, all studies that meet the inclusion criteria will be compiled and imported into Covidence (Veritas Health Innovation Ltd), which is a reference management software. Here, duplicate entries are eliminated. The eligibility of titles and abstracts will be screened by one reviewer under the supervision of another reviewer. Subsequently, full text will be individually evaluated by the 2 reviewers. Any discrepancies will be resolved through discussion or involvement of a third reviewer. The exclusion of full-text studies and the reason for exclusion will be reported in the final scoping review. The results of the search will be presented in a PRISMA (Preferred Reporting Items for Systematic Reviews and Meta-Analyses) flow diagram and fully reported in the final scoping review. As recommended by Joanna Briggs Institute, this scoping review will apply the PCC (participants, concept, context) framework.

#### Participants

The scoping review will consider studies that involve patients with multiple chronic conditions. Since multimorbidity and comorbidity have been applied interchangeably in the literature, both terms will be included. There are no restrictions on the types of diseases included, as long as they meet the criteria for chronic diseases, which are characterized by lasting for a prolonged period and often progressing over time. Research that focuses on individuals with at least 2 chronic diseases or studies that examine individuals with a single chronic disease at risk of developing another will be considered in this study.

#### Concept

This scoping review will consider studies that evaluate machine learning models applied on a population diagnosed with multimorbidity. This includes supervised learning methods, such as logistic regression, support vector machine, decision tree, random forest, or neural network.

#### Context

This scoping review will consider studies concerning either on the diagnosis, treatment, monitoring, or prediction of diseases in patients with multimorbidity.

#### Inclusion and Exclusion Criteria

Studies that examine patients with more than 1 chronic disease or individuals with a single chronic disease who are prone to developing another will be included in this study. A chronic disease is defined as a long-term health condition that necessitates complex and continuous medical care, which either can be mental or physical health conditions [1].

Due to the interchangeable use of the 2 terms multimorbidity and comorbidity in the literature, both terms will be considered in this study. Additionally, only studies using supervised learning algorithms will be considered. Supervised learning algorithms are trained on labeled data, allowing them to learn from examples with known input-output pairs.

This scoping review will consider all study types and aims to find published and unpublished studies. Furthermore, studies with full text and free full text will be included, since there is an interest in looking at their process, such as input data, methods, model, and outcome. Full-text studies published in all languages will be considered, provided there is an abstract available in English, French, Spanish, or German.

## Results

To ensure all relevant results are extracted from the identified studies, a template based on Aromataris et al [46] with extraction fields is adapted to suit our specific requirements. These can be seen in Textbox 1. For managing and screening references, Covidence will be used. For data extraction, Microsoft Excel (Microsoft Corp) will be applied. Data will be extracted by 2 reviewers, including information about the study population, method, medical application, and outcome.

**Overview of extraction fields for data retrieval from the identified studies. Inspired from Joanna Briggs Institute methodology guidance for scoping reviews [46].**

**Extraction fields**

- Authors
- Publication year
- Source of origin or country of origin
- Objective
- Study population or type of patient with multimorbidity and sample size
- Medical application
- Type of input data
- Type of algorithm
- Maturity of the algorithm
- Performance
- Validation of algorithm
- Outcomes
- Key findings that relate to review question

The data that have been extracted from the included studies will be presented in either tabular or diagrammatic format, depending on what is most suitable. These will also be supported by narrative descriptions. The key findings that relate to the review question will be analyzed thematically. Any emerging themes and subthemes will be depicted in a diagrammatic or alternative visual format that is appropriate.

## Discussion

### Principal Findings

Multimorbidity is a global challenge for the health care system since it is associated with increased mortality, reduced quality of life, and increased health care costs. With the increasing prevalence, the problem is only growing. Machine learning has the potential to address these challenges, as these methods can understand the patterns and factors associated with multimorbidity. This scoping review aims to explore and synthesize the current literature addressing the use of machine learning in the context of patients with multimorbidity.

Following the definition of a scoping study established by Arksey and O’Malley [47], this scoping review serves 2 primary objectives. It aims to compile and communicate research findings to policy makers, practitioners, and consumers. Besides that, it will also pinpoint any existing research gaps within the literature [47]. The available literature indicates ongoing research into the application of machine learning within the context of multimorbidity. Unfortunately, there is uncertainty regarding the type and quantity of evidence as well as potential knowledge gaps. To our knowledge, this may be the first scoping review in this field to uncover this.

The insights gained from this scoping review can be valuable for both researchers and health care practitioners. For researchers, understanding the current landscape of machine learning methods can provide a foundation for future studies by stimulating new research questions and help to identify research gaps. Health care practitioners can gather awareness of the current state of machine learning use in multimorbidity research. Overall, the findings from this scoping review can contribute to more precise interventions, ultimately improving patient outcomes.

### Limitations

Nevertheless, the scoping review may have some limitations regarding the search strategy. The scoping review follows the PCC framework, where including the last block (context) may potentially constrain the search outcomes. A potential solution is only to use the first 2 blocks (participants and concept), although this approach may introduce more noise into the search results. However, this might not pose an issue, as the last block (context) is comprehensive due to the included search words within the block.

A particular challenge in this field is the interchangeable use of the terms “multimorbidity” and “comorbidity” in the literature. To address this issue, the participant search block in the PCC framework contains synonyms for both terms. This ensures the retrieval of relevant studies and accounts for potential variations in terminology across the literature. Besides that, it also captures a broader spectrum of research related to patients with multiple chronic conditions. A disadvantage of this approach is the potential for more noise and irrelevant results.

Whether there is a need for quality assessment of the included studies can be discussed. Typically, this is not done in a scoping review, as the objective is to provide an overview of the evidence rather than a critically appraised and synthesized response to a specific question [48-50]. On the other hand, using tools such as the Prediction Model Risk of Bias Assessment, which is designed for systematic reviews, could provide insights into the quality of prediction models [51]. Being aware of this, this study will follow the typical approach for conducting scoping reviews, and thereby not conduct a quality appraisal of the included studies.

### Conclusions

The primary objective of this scoping review is to investigate the existing literature on machine learning in patients with multiple chronic conditions and potentially highlight research gaps. To our knowledge, this scoping review marks the initial exploration into the application of machine learning in multimorbidity research.

The outcome of the searches will be presented following the PRISMA-ScR checklist and flow diagram, which will provide a comprehensive overview of the search process, including the number of identified studies, duplicates removed, screened studies, excluded studies, and studies included in the review. A conclusive summary based on the results of the scoping review will be presented, and recommendations for future research will be suggested if any possible knowledge gaps are identified. The potential findings of the scoping review will be reported in an academic paper, which will be submitted to a peer-reviewed journal and presented at a national conference.

## Abbreviations

COPD: chronic obstructive pulmonary disease
PCC: participants, concept, context
PRISMA: Preferred Reporting Items for Systematic Reviews and Meta-Analyses
PRISMA-ScR: Preferred Reporting Items for Systematic Reviews and Meta-Analyses Extension for Scoping Reviews

## Data Availability

Data sharing is not applicable to this paper as no data sets were generated or analyzed during this study.
